# Oxidative Stress Signaling in Blast TBI-Induced Tau Phosphorylation

**DOI:** 10.3390/antiox10060955

**Published:** 2021-06-15

**Authors:** Chunyu Wang, Changjuan Shao, Li Zhang, Sandra L. Siedlak, James S. Meabon, Elaine R. Peskind, Yubing Lu, Wenzhang Wang, George Perry, David G. Cook, Xiongwei Zhu

**Affiliations:** 1 Department of Neurology, The Second Xiangya Hospital, Central South University, Changsha 410083, China; wangchunyu@csu.edu.cn; 2 Department of Pathology, Case Western Reserve University, Cleveland, OH 44106, USA; cxs708@case.edu (C.S.); lenzhangli@hotmail.com (L.Z.); sls7@case.edu (S.L.S.); yubing.lu@case.edu (Y.L.); wenzhang.wang@case.edu (W.W.); 3 Department of Endocrinology and Metabolism, Huashan Hospital, Fudan University, Shanghai 200240, China; 4 VA Puget Sound Health Care System, Seattle, WA 98108, USA; James64@uw.edu (J.S.M.); peskind@uw.edu (E.R.P.); dgcook@uw.edu (D.G.C.); 5 Department of Psychiatry and Behavioral Sciences, University of Washington, Seattle, WA 98115, USA; 6 Department of Biology, College of Science, University of Texas at San Antonio, San Antonio, TX 78229, USA; George.Perry@utsa.edu; 7 Departments of Medicine and Pharmacology, University of Washington, Seattle, WA 98195, USA

**Keywords:** blast, traumatic brain injury, tau phosphorylation, oxidative stress, ERK, JNK, p38, GSK3β

## Abstract

Traumatic brain injury caused by blast is associated with long-term neuropathological changes including tau phosphorylation and pathology. In this study, we aimed to determine changes in initial tau phosphorylation after exposure to a single mild blast and the potential contribution of oxidative stress response pathways. C57BL/6 mice were exposed to a single blast overpressure (BOP) generated by a compressed gas-driven shock tube that recapitulates battlefield-relevant open-field BOP, and cortical tissues were harvested at different time points up to 24 h after blast for Western blot analysis. We found that BOP caused elevated tau phosphorylation at Ser202/Thr205 detected by the AT8 antibody at 1 h post-blast followed by tau phosphorylation at additional sites (Ser262 and Ser396/Ser404 detected by PHF1 antibody) and conformational changes detected by Alz50 antibody. BOP also induced acute oxidative damage at 1 h post-blast and gradually declined overtime. Interestingly, Extracellular signal-regulated kinase (ERK) and c-Jun N-terminal kinase (JNK) were acutely activated in a similar temporal pattern as the rise and fall in oxidative stress after blast, with p38 showing a similar trend. However, glycogen synthase kinase-3 β (GSK3β) was inhibited at 1 h and remained inhibited for 24 h post blast. These results suggested that mitogen-activated protein kinases (MAPKs*)* but not GSK3β are likely involved in mediating the effects of oxidative stress on the initial increase of tau phosphorylation following a single mild blast.

## 1. Introduction

Traumatic brain injury (TBI) is a serious public health problem and major environmental risk factor for the later development of neurodegenerative disease including sporadic Alzheimer’s disease. An estimate of 10–20% of returning Iraq and Afghanistan veterans were found to have suffered at least a mild TBI (mTBI), the majority of which have been associated with exposure to explosive blasts (bTBI) [1]. An estimated 7.5–40% of these veterans have continued to report cognitive and post-concussive symptoms, including memory and cognitive deficits, months or even years after initial symptoms were resolved [2,3,4]. A history of TBI in older veterans was associated with a two-to-three fold increase in the risk of developing dementia among all races [5]. There is increasing concern that veterans with a history of blast traumatic brain injury (bTBI) have increased risk for developing chronic traumatic encephalopathy (CTE), a progressive neurodegenerative disorder affecting individuals exposed to head injuries, such as football players and boxers [6,7,8]. The symptomatology and neuropathology of CTE partially overlap with those of Alzheimer’s disease, as both disorders demonstrate neurological deficits, cognitive deficits, and dementia and are characterized by widespread tau pathologies composed of hyperphosphorylated tau proteins [9]. However, the understanding of the relationship between blast TBI and the development of CTE is still in its infancy, and the significance of tau neuropathology in blast-related mTBI is not currently resolved [6,7,8,10,11]. In addition, much remains to be elucidated about the relationship(s) of blast-induced aberrant phospho-tau expression and other important pathophysiological processes, including oxidative stress signaling, as reported by us and others [12,13,14].

Despite the subtle signs of injury, exposure to even a single mTBI could elicit long-term neuropathological changes many years after initial injury in human brains [15]. Blast TBI involves an axonal injury within 90 days after injury in veterans that remains persistent over 6–12 months [16] and that could be linked to impairment in executive function after TBI [17]. Accumulating case reports have found perivascular neurofibrillary tangles (NFTs) at the depths of the sulci in veterans with a history of blast exposure [18,19,20]. However, one recent study reported no tau pathology in five veterans exposed to a single blast event [21], although the possibility of the involvement of earlier but reversible tau alterations in the brains of these patients during the course of disease could not be ruled out. In vivo imaging technology makes it possible to study tau pathology in living patients, and this has revealed that up to 50% of veterans with a history of multiple blast exposure have shown excess cortical retention of tau ligands, demonstrating that tauopathy is present in a subgroup of veterans with blast TBI [7,22,23]. Clearly, there is an unmet need to understand the biological basis and mechanism of injury underlying blast TBI-induced neuropathological changes and neurological deficits.

Various animal models have been developed to study the effects of blast TBI and its underlying mechanism. Prior studies on the brains of blast TBI animal models found significantly elevated tau phosphorylation accompanying cognitive impairments [12,19,24]. For example, C57BL/6 mice demonstrated increased tau phosphorylation at S202 recognized by the CP-13 antibody and at the T181 site recognized by the AT270 antibody in the mouse brain two weeks after single-blast exposure [19]. Our prior studies also confirmed increased tau phosphorylation in these and additional sites (e.g., S212, T214, T231, and S396), along with increased tau cleavage in various brain areas 24 h after a single-blast that lasted for 30 days [12]. Increased tau phosphorylation at multiple sites was also reported several weeks after double or repetitive blast exposure [25,26]. Some studies have suggested that increased tau phosphorylation at specific sites may, in fact, be specific to the type and severity of blast exposure [27,28]. However, limited studies have been carried out to assess early changes after blast, which we consider to be of particular importance in the understanding of the underlying molecular mechanisms that may inform better intervention to prevent the progression of the disease. In this study, we aimed to determine changes in initial tau phosphorylation after exposure to a single mild blast and the potential contribution of oxidative stress and stress response pathways including the three mitogen-activated protein kinases (MAPK) pathways [i.e., the extracellular signal-regulated kinase (ERK), c-Jun N-terminal kinase (JNK) and p38 pathways] and the glycogen synthase kinase-3 β (GSK3β) pathway.

## 2. Materials and Methods

### 2.1. Animals and Treatment

Three-month-old, male C57Bl/6J mice (*n* = 42) were used for this study. All mice were socially housed in groups of 3–4 with ad libitum food and water access in an Association for Assessment and Accreditation of Laboratory Animal Care (AAALAC)-accredited specific antigen-free (SPF) facility synchronized to a standard 12/12 h day/night cycle. All experiments were conducted in accordance with the National Institutes of Health Guide for the Care and Use of Laboratory Animals. Experiment group sample sizes, consisting of at least *n* = 5 blast and *n* = 5 sham for each timepoint examined in the study, were based on our prior work examining the development of nascent tauopathy [12]. Our established murine mild blast TBI model was previously characterized and reported [12]. Briefly, animal anesthesia was induced with brief 5% isoflurane exposure in oxygen at a rate of 1 L/min using a nonrebreathing apparatus. The induced mice were then maintained with 2.5% isoflurane anesthesia using a flexible custom nosecone while they were positioned with their dorsal aspect lying against a rigid gurney such that their ventral aspect (e.g., abdomen) was oriented towards the oncoming overpressure wave. Head and body motions caused by exposure to blast overpressure were minimized by the secure mounting of arms and legs above the wrist and ankle joints, respectively, as well as the torso using plastic ties to immobilize the mice against the support gurney. Non-blasted sham control mice were yoked with blast mice, similarly mounted into the shock tube, and held under anesthesia for the same amount of time.The blast overpressure waves used in these experiments had a peak static pressure of 19 ± 0.4 pounds per square inch (p.s.i.), a positive phase duration of 6.2 ± 0.08 msec (mean ± SEM, each), and a resulting impulse of ~32.6 psi * milliseconds. All animals survived for the duration of their respective study timepoint. At the conclusion of study timepoints, animals were euthanized by lethal sodium pentobarbital injection and dissected. Tissues were immediately frozen in liquid nitrogen and kept at −80 °C until used.

### 2.2. Western Blot

Mouse cortical tissues were carefully dissected out and homogenized with a radioimmunoprecipitation assay (RIPA) lysis buffer plus protease inhibitor mixture (5892791001/4906837001, Roche, Penzberg, Germany). Homogenates were centrifuged at 14,000 rpm for 20 min, and the supernatants collected and the protein level was determined using a bicinchoninic acid (BCA) assay (23225, Thermo Fisher Scientific, Waltham, MA, USA). Equal amounts of total protein extracts were resolved by sodium dodecyl sulphate–polyacrylamide gel electrophoresis (SDS-PAGE) and transferred to Immobilon-P (IPVH00010, Millipore, Temecula, CA, USA). Following blocking with 10% nonfat milk, appropriate primary and secondary antibodies were applied, and the blots were developed with Immobilon Western Chemiluminescent horseradish peroxidase (HRP) substrate (WBKLS0500, Millipore, Temecula, CA, USA).

### 2.3. Antibodies

The primary antibodies used in this study included rabbit anti-4-HNE (HNE11-S, Alpha diagnostics, San Antonio, TX, USA), heme-oxygenase 1 (Enzo, Farmingdale, NY, USA), nitrotyrosine (10189540, Cayman Chemical, Ann Arbor, MI, USA), pGSK3β Ser9 (9322, Cell Signaling Technology, Danvers, MA, USA), GSK3β (32391, Abcam, Cambridge, MA, USA), pERK1/2 (9106, Cell Signaling Technology, Danvers, MA, USA), total ERK1/2 (9102, Cell Signaling Technology), pJNK (9251, Cell Signaling Technology), total JNK (9252, Cell Signaling Technology), p38 (Cell Signaling Technology), pp38 (Cell Signaling Technology), and actin (clone C4, EMD Millipore, Burlington, MA, USA). Antibodies specific for phosphorylated tau at sites Ser 262 (ab4856, Abcam), Ser202/Thr205 (AT8, Invitrogen, Waltham, MA, USA), and Ser396/Ser404 (PHF1, gift of Sharon Greenberg and Peter Davies), as well as the conformational tau-specific antibody Alz50 (gift of Peter Davies, Albert Einstein College of Medicine, Bronx, NY, USA) and total tau (tau 5, Invitrogen), were used. Secondary antibodies used in this study included an anti-mouse/rabbit HRP-linked secondary antibody (7076 and 7074, Cell Signaling Technology, Danvers, MA, USA) and a goat anti-mouse or goat anti-rabbit (AP124 or AP132, Millipore, Temecula, CA, USA) peroxidase-conjugated antibody.

### 2.4. Image Acquisition and Statistical Analysis

Western blot images were captured using either a GE Amersham Imager 600 (GE Amersham, Boston, MA, USA) or film, and they were quantified with Image J 2.0.0 (National Institutes of Health, Bethesda, MD, USA). Images were converted to 8-bit, and the background was subtracted. Measurements were set as follows: pick area, mean grey value, and integrated density, and images inverted. Using a freehand tool, the area encompassing the bands was outlined and values were obtained. Specifically, densitometric values were obtained for all phosphorylated tau bands detected by each antibody, which were quantified relative to total tau level for each individual mouse (tau-5). One major band was detected for both HO-1 and 4-HNE, but multiple bands were detected for 3-NT (see Supplementary Materials). All bands labeled for 3-NT from 31 kDa to the top of the blot were detected with Image J using the gel tool function, and the sum of the peaks was measured. The levels of 3-NT, 4-HNE, and HO-1 were quantified relative to actin. All bands specific for phosphorylated GSK3β, JNK, ERK, and p38 were compared to total GSK3β, JNK, ERK, and p38, respectively. Statistical analysis was performed using GraphPad Prism 8 software 8.4.0(455) (GraphPad Software, Inc. San Diego, CA, USA). Data were analyzed by a Student’s *t*-test to compare the difference between the bTBI and sham mice. All the data were expressed as means  ±  SEM, and are shown relative to the mean of the sham 1 h group. *p* values are indicated by asterisks (*** *p* < 0.001; ** *p* < 0.01; * *p* < 0.05).

## 3. Results

### 3.1. Effects of a Single Mild BOP Exposure on Tau Phosphorylation

**Figure 1:**
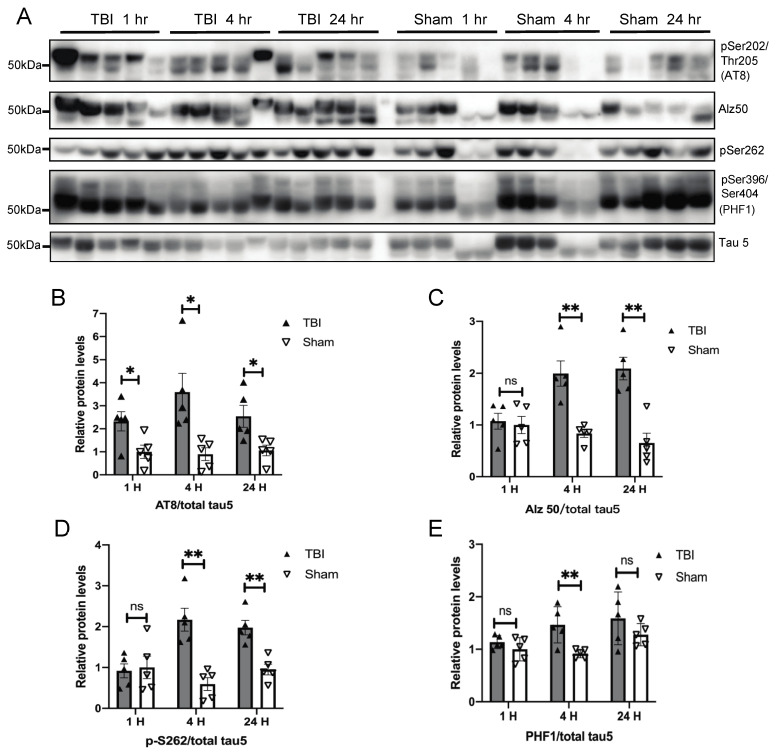
A single blast overpressure (BOP) caused increased tau phosphorylation in the mouse brain. (**A**) Representative Western blots demonstrated the early elevation of phospho-tau (Ser202/Thr205 detected by AT8, pS262, and Ser396/Ser404 detected by PHF1 antibody and pathological conformation changes of tau (Alz50) in the cortex after blast (*n* = 5/group). Tau-5 was probed to detect total tau. (**B**–**E**) Quantification analysis of AT8 (**B**), Alz50 (**C**), pS262 (**D**), and PHF1 (**E**) normalized to total tau detected b they tau 5 antibody. (Data are means ± SEM, * *p* < 0.05, ** *p* < 0.01; ns, non-significant). TBI: traumatic brain injury.

We previously reported that 24 h after exposure to a single mild blast overpressure (BOP), wild-type C57BL/6 mice demonstrated increased tau phosphorylation in the brain that lasted for 30 days [12]. To investigate the mechanism underlying increased tau phosphorylation, we focused on short-term changes in the cortex of exposed mice in the current study. Cortical tissues from BOP and sham controls were examined at 1, 4, and 24 h post exposure for tau phosphorylation by western blot using several well-characterized tau antibodies (Figure 1A). Significantly increased tau phosphorylation at Ser202/Thr205 detected by the AT8 antibody was found 1 h post-exposure, which lasted until 24 h in the BOP mice (Figure 1B). Significantly increased phosphorylation at S262 and Ser396/Ser404 detected by PHF1 antibody was also found at 4 h post-exposure in BOP mice (Figure 1D,E). Interestingly, the significantly increased immunoreactivity of Alz50 was also found in the brain of BOP mice at 4 h post-exposure and lasted until 24 h (Figure 1C). Through Western blot, Alz50 has been shown to be increased in various neuropathological conditions [29,30], and it is considered specific for pathological tau conformation in Alzheimer’s disease [31].

### 3.2. Effects of a Single Mild BOP Exposure on Oxidative Stress

**Figure 2:**
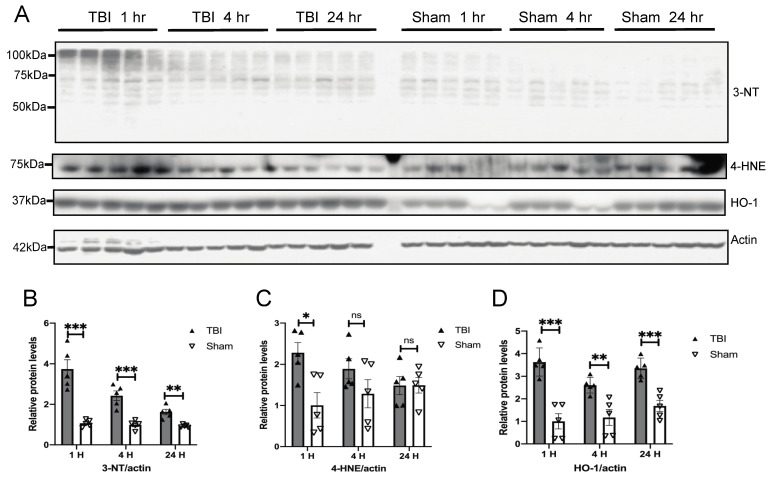
BOP caused increased oxidative stress in the mouse brain. (**A**) Representative Western blots demonstrated the early induction of oxidative stress markers in the mouse brain after blast (*n* = 5/group). Actin was probed as an internal loading control. (**B**–**D**) Quantification analysis of protein oxidative marker 3-nitrotyrosine (3-NT) (**B**), lipid peroxidation marker 4-hydroxynonenal (4-HNE) (**C**), and antioxidant hemeoxygenase-1 (HO-1) (**D**) normalized to actin levels. (Data are means ± SEM, * *p* < 0.05, ** *p* < 0.01, *** *p* < 0.001; ns, non-significant).

To determine whether oxidative stress is involved [32], we examined the temporal expression of oxidative stress markers in the brain of BOP and sham control mice following BOP exposure (Figure 2A). The Western blot for 3-nitrotyrosine (3-NT), a marker for oxidative damage to proteins, revealed multiples bands with significantly increased immunoreactivity in the brain of BOP mice at 1 h post-exposure, which gradually decreased but still remained significantly elevated at 24 h compared to that of the sham control mice (Figure 2A,B). Similarly, the Western blot analysis of 4-hydroxynonenal (4-HNE), a marker for lipid peroxidation, also revealed one major band that was significantly elevated at 1 h post-exposure that gradually declined over time (Figure 2C). The expression of hemeoxygenase-1 (HO-1), an antioxidant enzyme, was significantly elevated in the brain of BOP mice at 1 h post-exposure and remained significantly elevated throughout 24 h compared to sham treated mice (Figure 2D).

### 3.3. Effects of a Single Mild BOP Exposure on Tau Kinases

**Figure 3:**
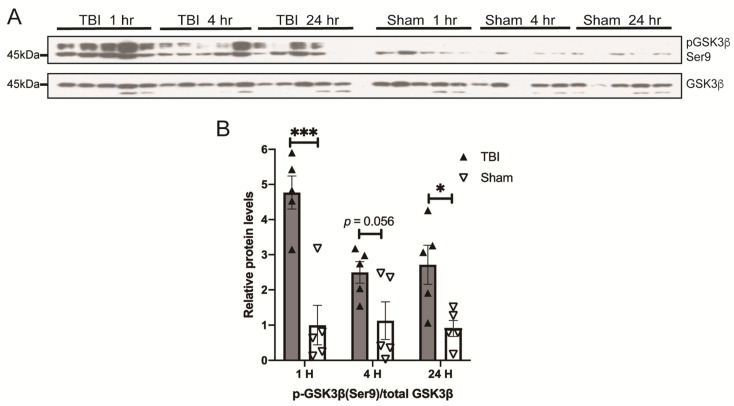
BOP caused the inhibition of glycogen synthase kinase-3β (GSK3β) in the mouse brain. Representative Western blots (**A**) and quantification analysis (**B**) revealed the inhibition of GSK3β after blast (*n* = 5/group). (Data are means ± SEM, * *p* < 0.05, *** *p* < 0.001).

Tau can be phosphorylated by multiple kinases, and GSK3β is the major one implicated in the phosphorylation of tau in AD [33]. GSK3β activity is inhibited by phosphorylation at Ser9. To determine whether GSK3β activation is involved in blast-induced tau phosphorylation, GSK3β phosphorylation at Ser9 was measured with a Western blot of the pGSK3β (Ser9) antibody (Figure 3A). There was no change in the GSK3β level between BOP and sham mice. Interestingly, significantly elevated levels of GSK3β phosphorylated at Ser9 were found in BOP mice as early as 1 h post-exposure, which remained elevated throughout 24 h after exposure, suggesting that GSK3β is likely inhibited by BOP exposure (Figure 3B).

**Figure 4:**
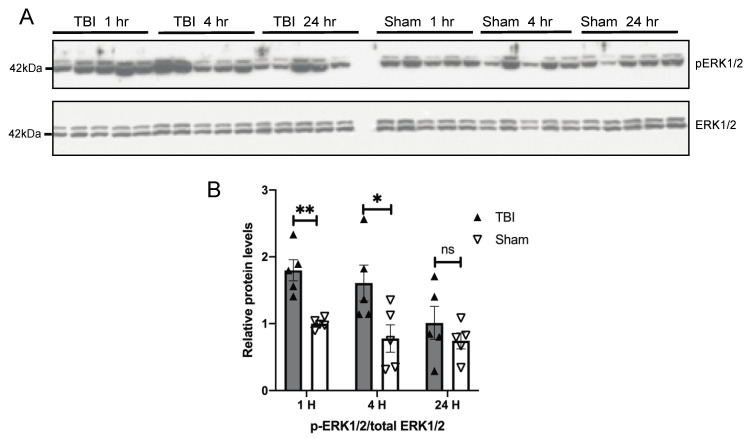
BOP caused the activation of extracellular signal-regulated kinase (ERK) in the mouse brain. Representative Western blots (**A**) and quantification analysis (**B**) revealed the activation of ERK after blast (*n* = 5/group). (Data are means ± SEM, * *p* < 0.05, ** *p* < 0.01; ns, non-significant).

**Figure 5:**
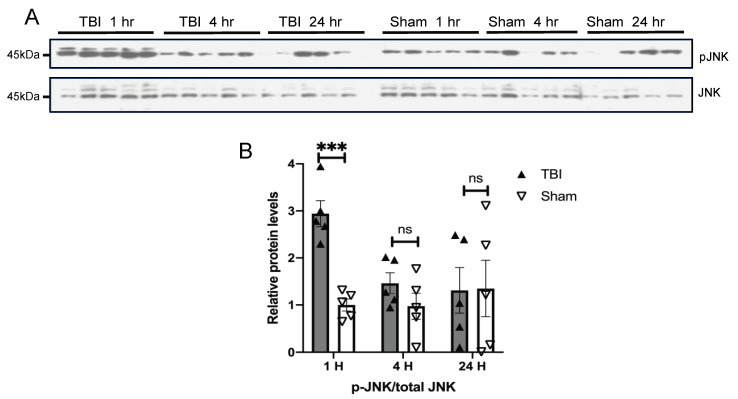
BOP caused the activation of c-Jun N-terminal kinase (JNK) in the mouse brain. Representative Western blots (**A**) and quantification analysis (**B**) revealed the activation of JNK after blast (*n* = 5/group). (Data are means ± SEM, *** *p* < 0.001; ns, non-significant). pJNK: phospho-JNK.

**Figure 6:**
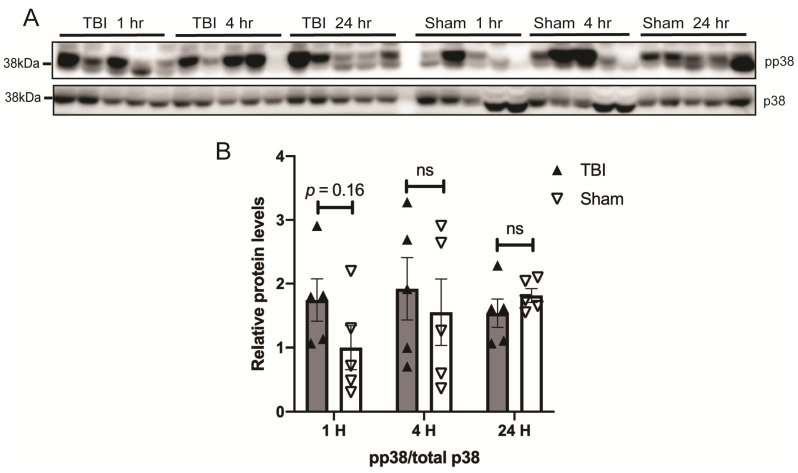
BOP caused a trend toward the activation of p38 in the mouse brain. Representative Western blots (**A**) and quantification analysis (**B**) revealed the activation of p38 after blast (*n* = 5/group). (Data are means ± SEM; ns, non-significant).

Given that BOP caused oxidative stress in the brain [32] and the mitogen-activated protein kinase pathways are the major signal transduction pathways that are activated by extracellular stimuli including oxidative stress [34], we next examined the temporal pattern of activation of MAPKs in the brain of BOP and sham control mice. A Western blot analysis revealed a nearly two-fold significant increase in the ratio of phosphorylated ERK/total ERK in the brain of BOP mice at 1 h post-exposure, which remained elevated at 4 and 24 h post-exposure, although did not reach significance after 24 h post-exposure (Figure 4). JNK was also significantly activated in the BOP mice at 1 h post-exposure. where the phosphorylated JNK/total JNK ratio was around 3-fold higher than that of sham control mice (Figure 5). However, JNK phosphorylation/activation in BOP mice quickly returned to the basal level at 4 h and became even lower at 24 h compared to sham control mice, although it did not reach significance (Figure 5). The p38 activation assessed by the level of the phosphorylated p38/total p38 ratio revealed a trend towards activation in the BOP mice at 1 h post-exposure and became indistinguishable with the sham control mice at 4 h post-exposure and beyond (Figure 6). 

## 4. Discussion

By focusing on the acute changes following a single mild BOP exposure, this study provides evidence that support the notion of a likely involvement of the activation of the MAPK pathways, but not the GSK3β pathway, in response to BOP-induced oxidative stress in the initial phosphorylation of tau proteins at multiple sites. This conclusion is based on the following observations: (1) a single mild blast led to significantly increased tau phosphorylation at AT8 sites as early as 1 h post-exposure, followed by increased phosphorylation at other sites such as Ser262, which resulted in pathological conformational changes detected by Alz50; (2) a single mild blast caused significant oxidative damage to proteins (i.e., 3-NT) and lipids (i.e., 4-HNE), which elicited long lasting antioxidant responses (i.e., HO-1 induction) concurrent to tau phosphorylation; (3) a single mild blast induced the acute activation of the ERK and JNK, as well as a trend towards the modest activation of the p38 pathways at 1 h post-exposure, which returned to control levels at 24 h post-exposure; and (4) a single mild blast led to the immediate and long-lasting inhibition of GSK3β kinase. These findings could have important implications in the understanding of mechanisms underlying the development of tau pathology in blast-TBI models and patients.

Tau alteration and deposition in the brain is a common feature of many neurodegenerative diseases including Alzheimer’s disease and CTE. More specifically, neurofibrillary and glial tangles containing phosphorylated tau have been found in the brains of military veterans with blast-related TBI [18,19,20]. Increased tau phosphorylation has been consistently demonstrated in the brains of animals exposed to blasts from 24 h to many months post-exposure [12,19,24,25,26]. In this study, we found increased tau phosphorylation at multiple sites 24 h after blast, which was consistent with prior studies. Intriguingly, we found increased tau phosphorylation at S202/T205 recognized by the AT8 antibody as early as 1 h after blast and increased phosphorylation at additional sites such as S262 and S396/S404 at 4 h after blast. This suggests that increased tau phosphorylation after blast is a very early event. However, it remains to be determined whether this specific temporal sequence of tau phosphorylation at different sites (e.g., initial phosphorylation at the AT8 site followed by phosphorylation at additional sites) is a treatment-specific phenomenon and/or of any functional significance. It has been demonstrated that tau hyperphosphorylation, cleavage, and conformational changes are critical for the formation of helical filament and tau aggregation [35]. Notably, we found increased Alz50 immunoreactivity 4 h after blast, which remained elevated at 24 h. The Alz50 antibody recognizes a specific tau conformation involving the juxtaposition of N- and C-termini stabilized by its incorporation into a structurally ordered tau filament that is considered an early pathological change during the formation of NFTs [36]. Thus, this finding provides additional evidence for the notion that a single blast exposure could set the stage for later NFT formation.

Concurrent to increased tau phosphorylation following blast, our study clearly demonstrated that a blast induced acute oxidative damage to both proteins and lipids in the brain 1 h after blast, which gradually declined over time until 24 h. Oxidative stress is an early and prominent feature in the brain of patients with Alzheimer’s disease and plays a critical role in tau hyperphosphorylation and the formation of tau pathology [37,38]. For example, 4-HNE modification to phospho-tau is essential in the formation of the pathological conformation that defines the Alz50 epitope and facilitates the formation of tau filaments [39]. Indeed, in this study, we found increased 4-HNE induction as early as 1 h post-BOP exposure, preceding the formation of the Alz50 epitope. Additionally, various molecules that induce oxidative stress, such as acrolein and homocysteine, increase tau phosphorylation both in vitro and in vivo via the activation of tau kinases through downstream stress-activated signaling pathways [40,41]. A large number of kinases and phosphatases are involved in the regulation of tau phosphorylation. We chose to focus on MAPKs and GSK3β in the current study because of their involvement in oxidative stress response. Interestingly, it appears that these tau kinases were differentially impacted in our study: ERK and JNK were acutely activated along with a trend towards the activation of p38 in a similar temporal pattern as the rise and fall in oxidative stress after blast; in contrast, GSK3β was inhibited, as evidenced by increased phosphorylation at the Ser9 inhibitory site at the same time of the oxidative stress increase, and remained inhibited throughout 24 h after blast. While both MAPKs and GSK3β can phosphorylate multiple sites in tau and are involved in tau phosphorylation in AD and other tauopathies [42], these data suggest that MAPKs, but not GSK3β, were likely involved in mediating the effects of oxidative stress on the initial increase of tau phosphorylation following a single mild blast in our study. It should be noted that the activation of MAPKs was relatively acute compared to longer-lasting tau phosphorylation, suggesting that changes in tau phosphatases may also be involved in later stages. In this regard, it was recently reported that the expression of protein phosphatase 2A-Bα was significantly reduced in the brains of rodents 15 days after an explosive-driven double blasts [26]. It was somewhat surprising to find that GSK3β was inhibited in our study given the major role of GSK3β activation in many tauopathies. Nevertheless, this finding was consistent with another study that demonstrated an increased phosphorylation of GSK3β at serine 9 starting at one day and persisted until at least six weeks after exposure to a single mild pulse shock wave, suggesting that the acute and long-lasting inhibition of GSK3β after a single blast may be common [43]. However, a prior study found GSK3β activation three weeks after repetitive blast exposure [25]. It is therefore possible that GSK3β may be activated in repetitive blast exposure, which likely requires additional stressors and could become relevant in a more complicated condition. In addition to tau phosphorylation, it is known that the activation of the MAPK pathways following oxidative stress could induce the expression of antioxidant enzymes and heat shock proteins including HO-1 [34]. Indeed, we found an induction of HO-1 around the same time as the activation of the MAPK pathways. Consistently, prior studies have reported the induction of SOD2, another antioxidant enzyme, after BOP exposure [12]. It appears that these antioxidant responses were effective in restoring redox balance in the brain, as oxidative damage to proteins and lipids gradually declined overtime and almost returned to the basal level at 24 h. Therefore, our findings suggest that exposure to a single mild BOP elicits an antioxidant response through MAPK activation that could effectively control oxidative damage but leave longer-lasting effects through increased tau phosphorylation.

## 5. Conclusions

In conclusion, our study demonstrated acute oxidative stress accompanied by increased tau phosphorylation after a single mild blast and suggested that mitogen-activated protein kinases (MAPKs) but not GSK3β are likely involved in mediating the effects of oxidative stress on the initial increase of tau phosphorylation.

## Data Availability

The data presented in this study are available on request from the corresponding author.

## Supplementary Materials

The following are available online at https://www.mdpi.com/article/10.3390/antiox10060955/s1, Figure S1: Original Western blot file.
