# Supplementary material for: Oxidative Stress Signaling in Blast TBI-Induced Tau Phosphorylation
Source: Antioxidants (Basel). 2021 Jun 15;10(6):955. doi: 10.3390/antiox10060955 (PMC8232162; doi:10.3390/antiox10060955)
Supplement: Supplementary file 1 [file antioxidants-10-00955-s001.zip › antioxidants-1187271-supplementary.pdf]

Oxidative stress signaling in blast TBI-induced tau Phosphorylation

Chunyu Wang 1,2, Changjuan Shao 2, Li Zhang 2, 3, Sandra L. Siedlak 2, James S. Meabon 4,5, Elaine R. Peskind 4,5, Yubing Lu 2, Wenzhang Wang 2, George Perry 6, David G. Cook 4,5,7 and Xiongwei Zhu 2,*
.

1 Department of Neurology, the second Xiangya Hospital, Central South University, Changsha, Hunan, People's Republic of China; wangchunyu@csu.edu.cn
2 Department of Pathology, Case Western Reserve University, Cleveland, OH, USA; cxs708@case.edu, yubing.lu@case.edu; sls7@case.edu, wenzhang.wang@case.edu, xiongwei.zhu@case.edu
3 Department of Endocrinology and Metabolism, Huashan Hospital, Fudan University, Shanghai, People's Republic of China; lenzhangli@hotmail.com,
4 VA Puget Sound Health Care System, Seattle, WA, USA; James64@uw.edu, peskind@uw.edu, dgcook@uw.edu
5 Department of Psychiatry and Behavioral Sciences, University of Washington, WA, USA
6 Department of Biology, College of Science, University of Texas at San Antonio, San Antonio, Texas, USA; george.perry@utsa.edu
7 Departments of Medicine and Pharmacology, University of Washington, WAQ, USA
* Correspondence: xiongwei.zhu@case.edu; Tel.: +1-216-368-5903

Actin

TBI 1hr | TBI 4hr | TBI 24hr | Sham 1 hr | Sham 4hr | Sham 24hr

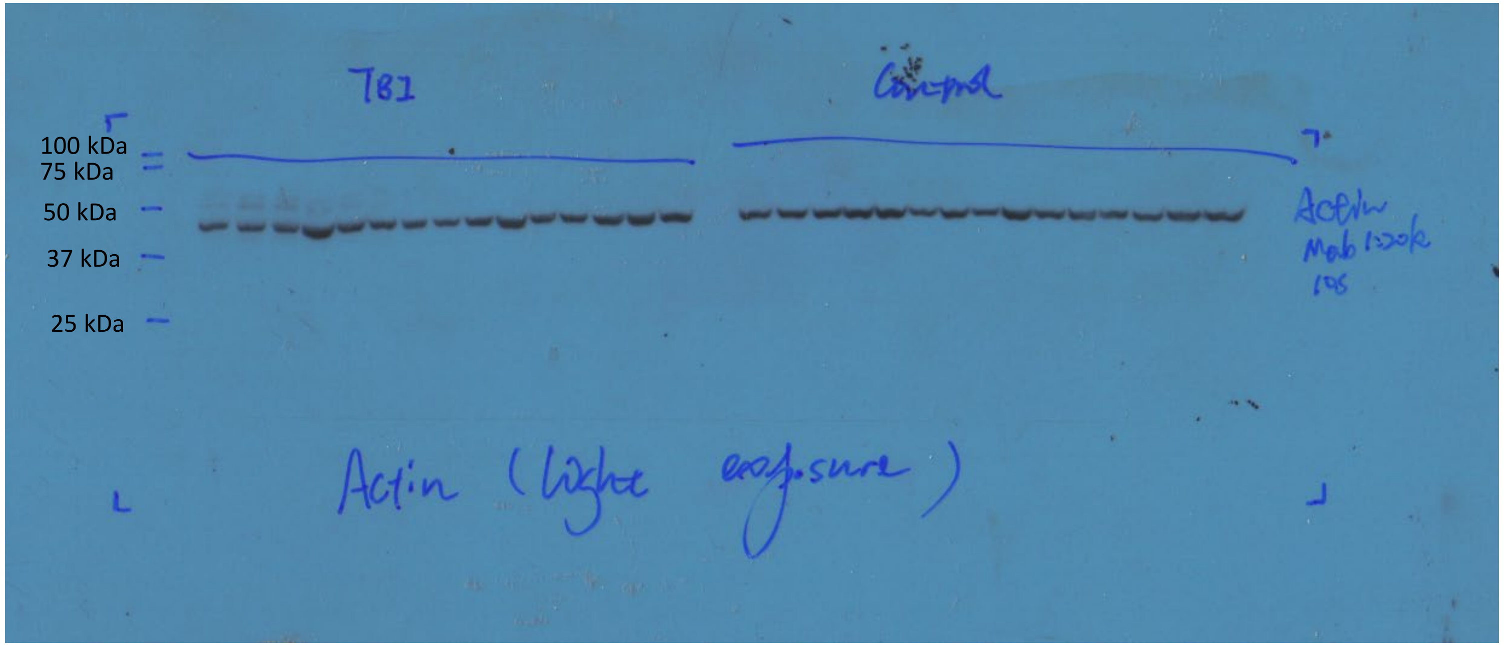

TBI 1hr | TBI 4hr | TBI 24hr | Sham 1 hr | Sham 4hr | Sham 24hr

pGSK3β ser9

Phospho-JNK

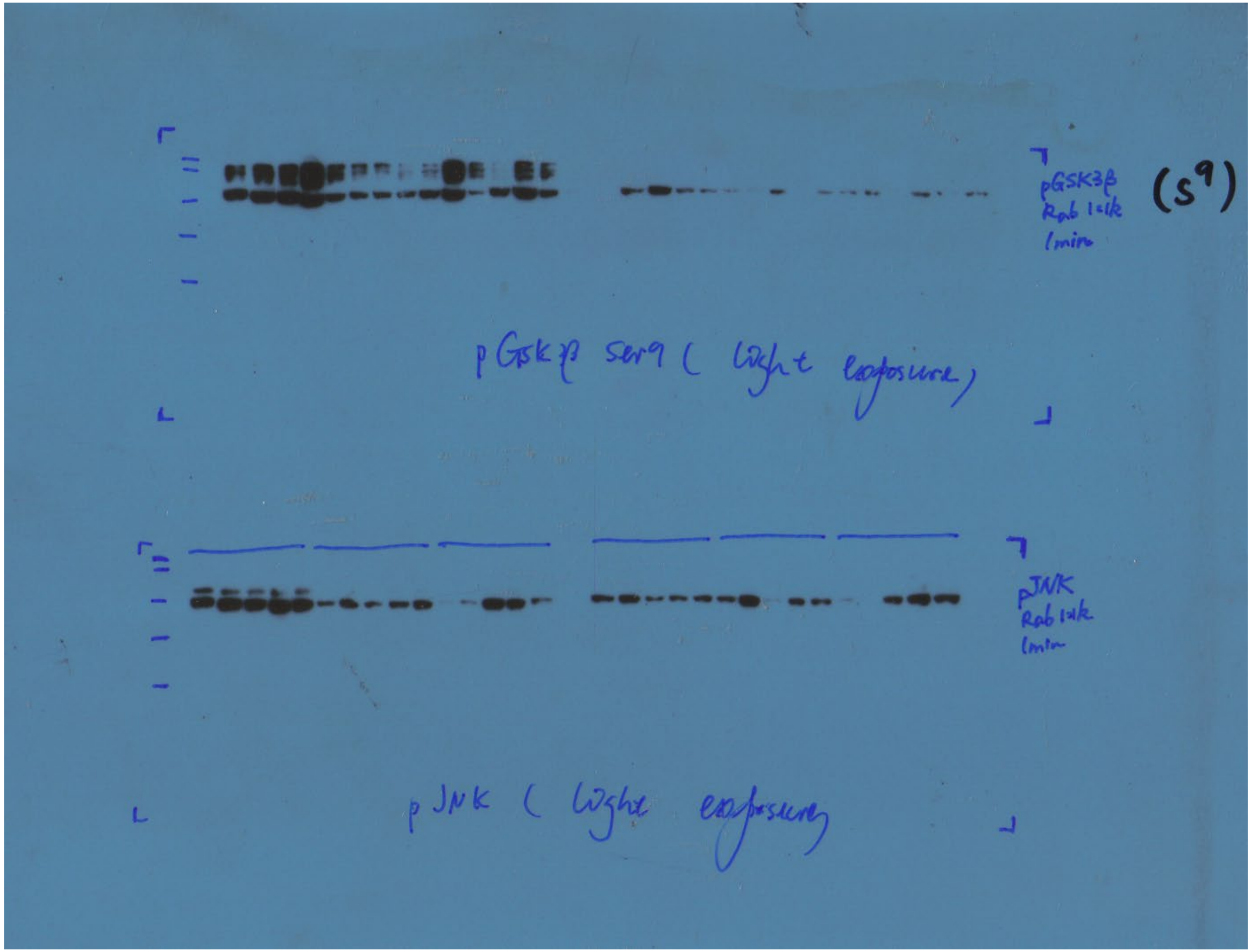

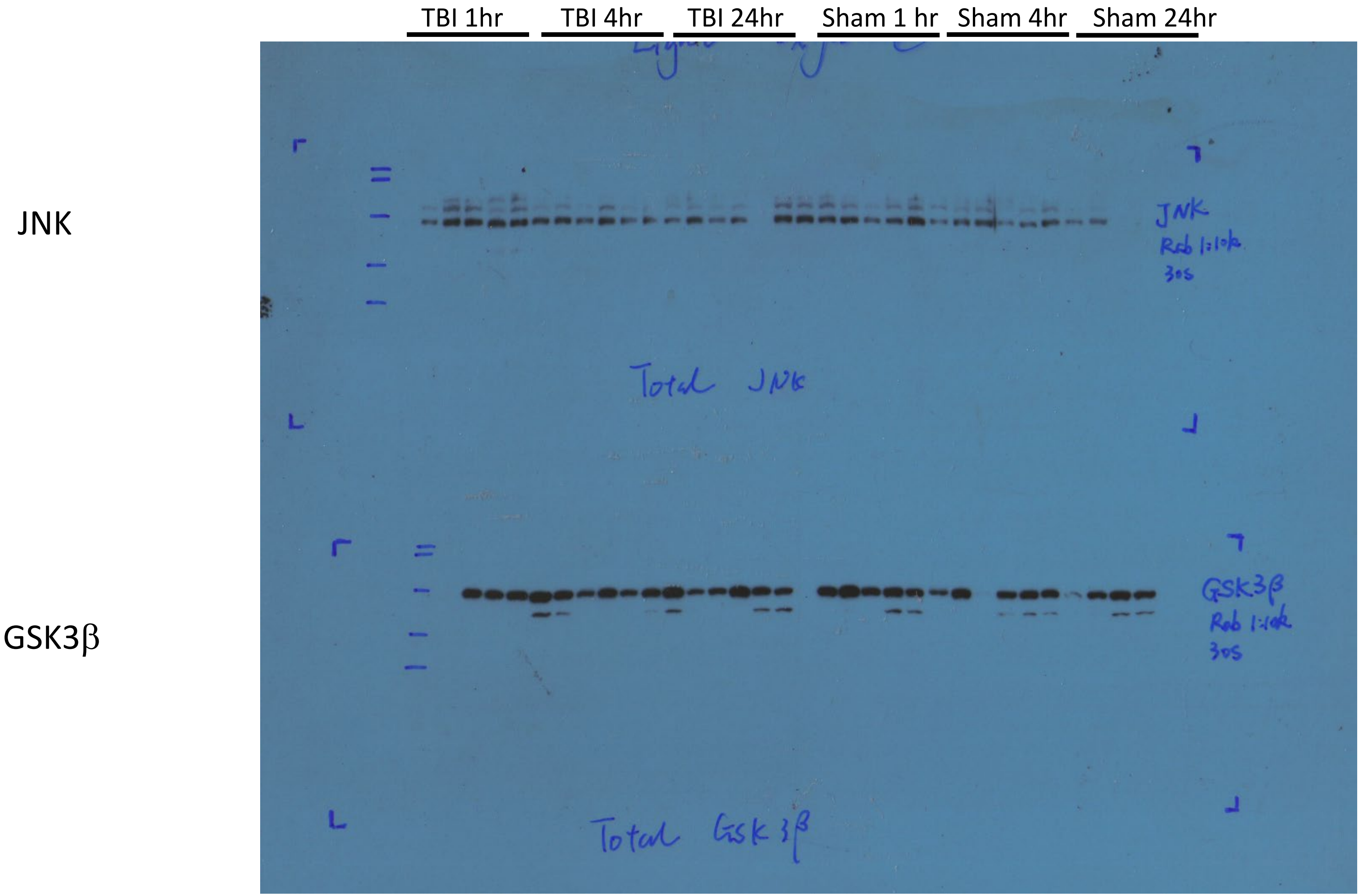
TBI 1hr
TBI 4hr
TBI 24hr
Sham 1 hr
Sham 4hr
Sham 24hr
JNK
GSK3β
Total JNK
Total GSK3β

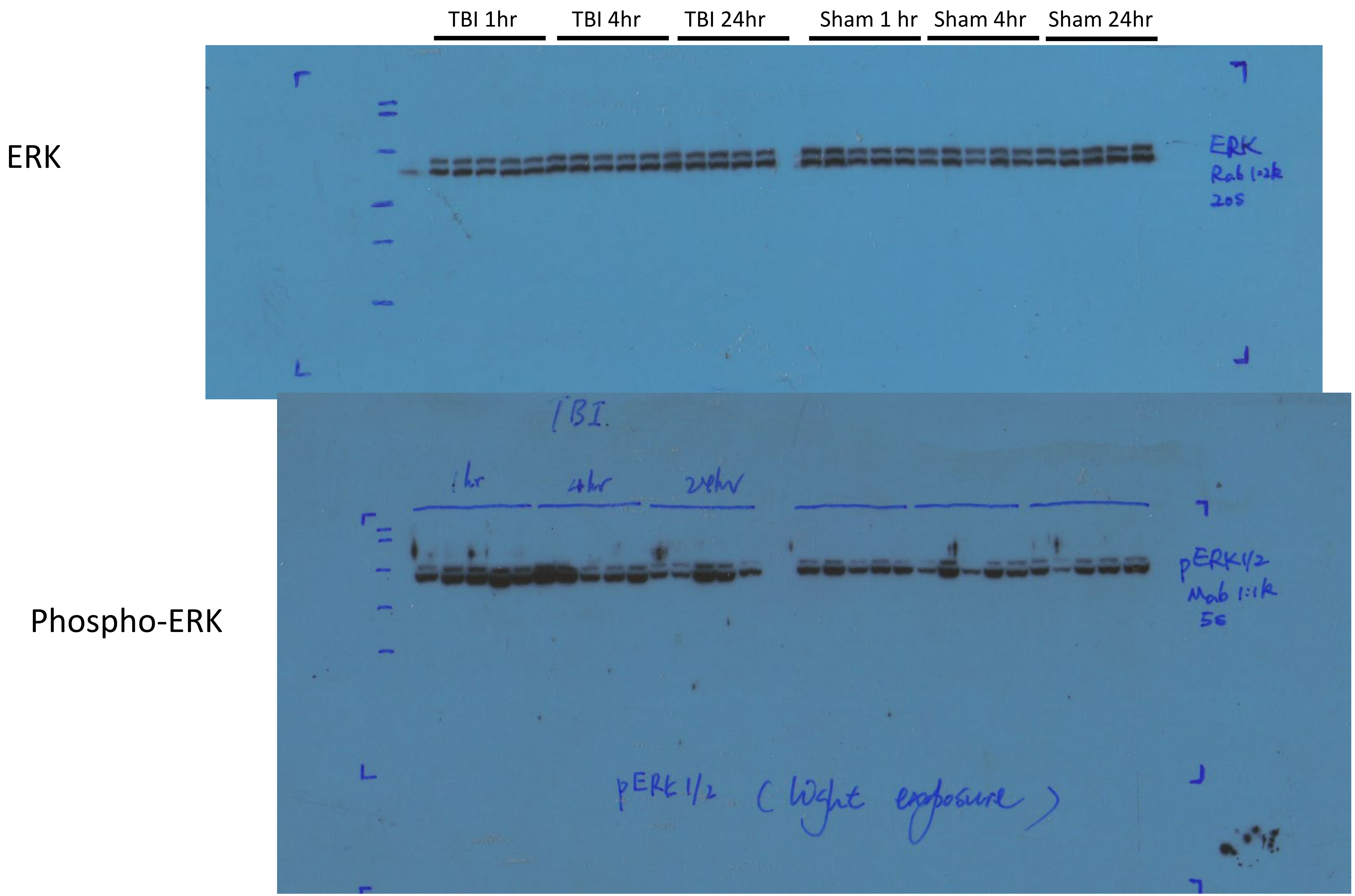
TBI 1hr
TBI 4hr
TBI 24hr
Sham 1 hr
Sham 4hr
Sham 24hr
ERK
ERK
Rab 1:2k
20S
Phospho-ERK
TBI
1hr
4hr
24hr
pERK1/2
Mab 1:1k
5S
pERK1/2 (light exposure)

Nitrotyrosine

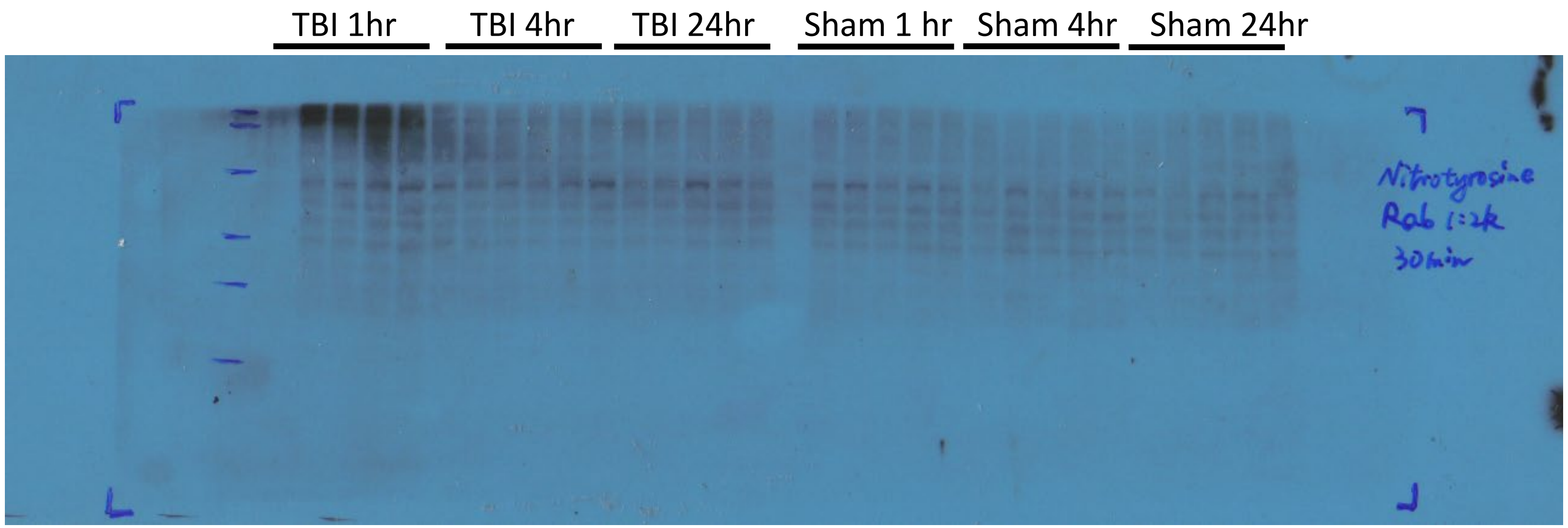

Tau5

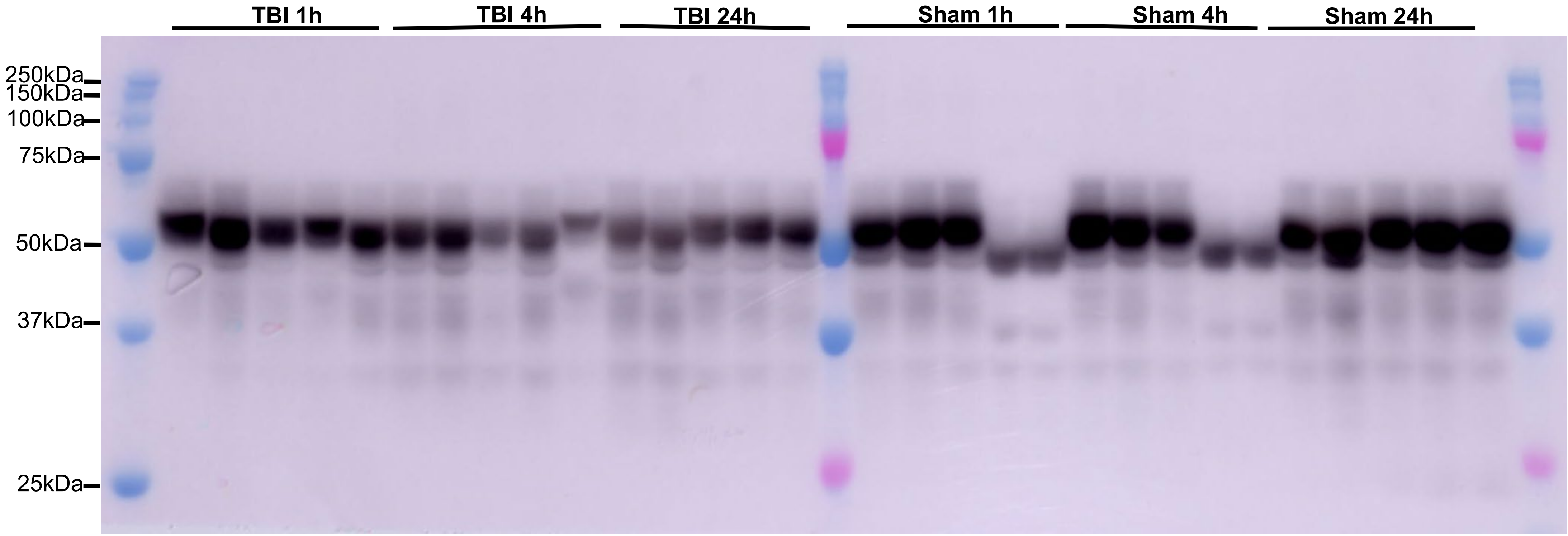
TBI 1h
TBI 4h
TBI 24h
Sham 1h
Sham 4h
Sham 24h
250kDa
150kDa
100kDa
75kDa
50kDa
37kDa
25kDa

AT8

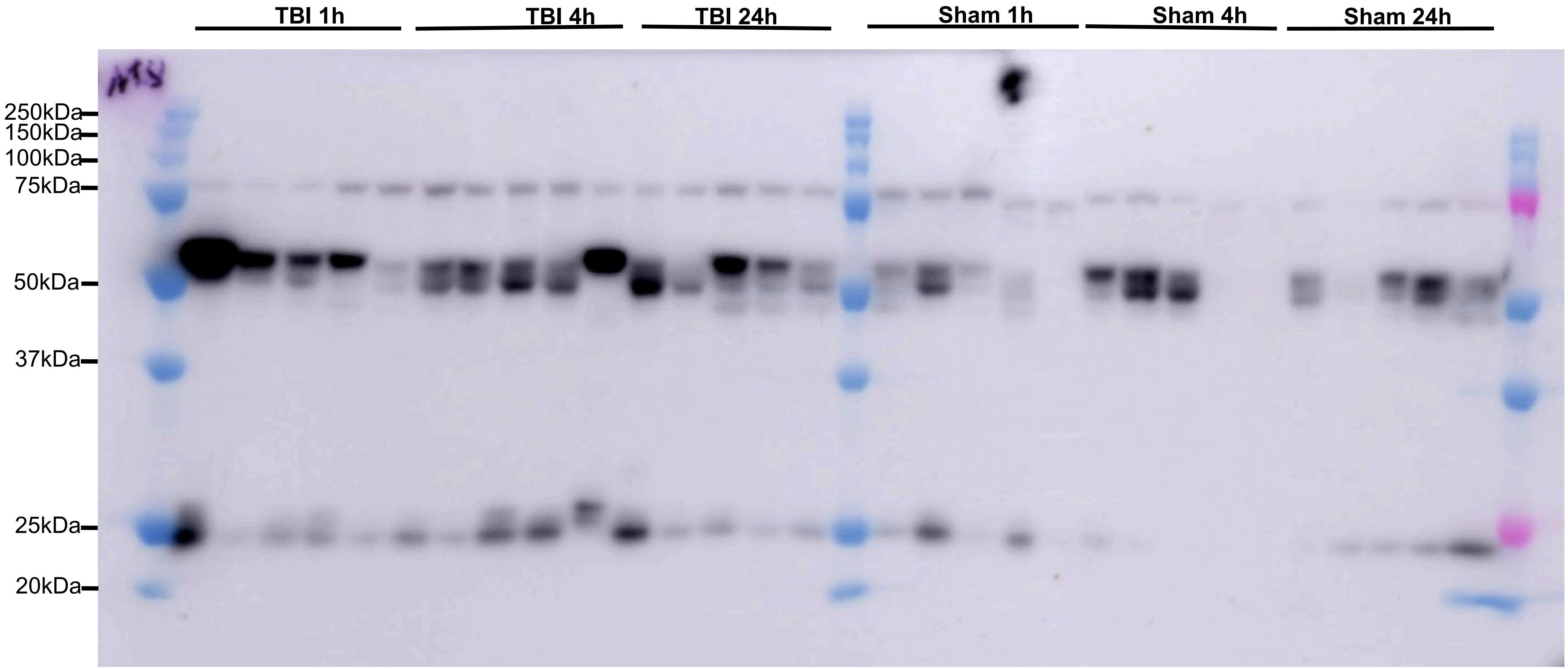
TBI 1h
TBI 4h
TBI 24h
Sham 1h
Sham 4h
Sham 24h
250kDa
150kDa
100kDa
75kDa
50kDa
37kDa
25kDa
20kDa

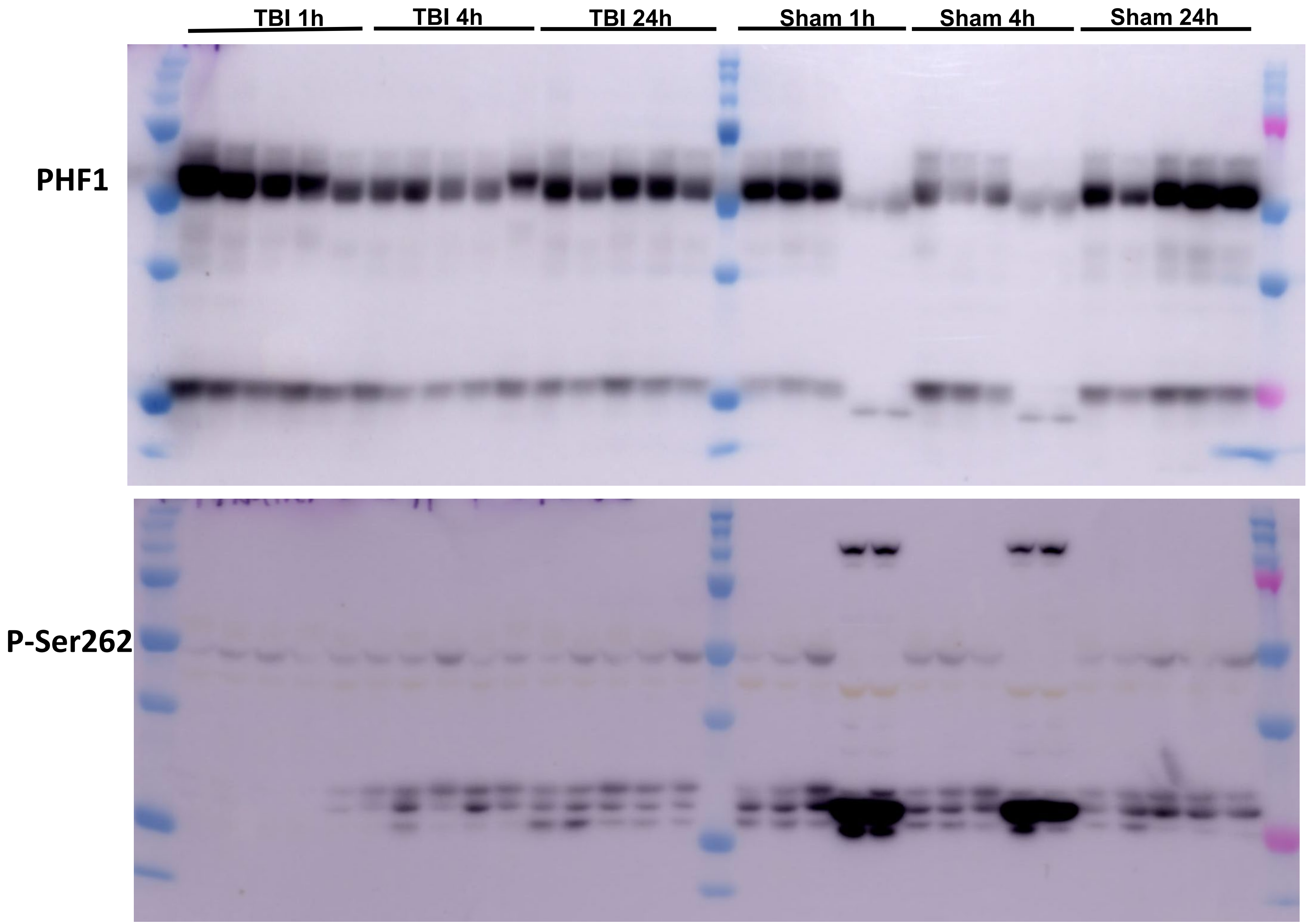
TBI 1h
TBI 4h
TBI 24h
Sham 1h
Sham 4h
Sham 24h
PHF1
P-Ser262

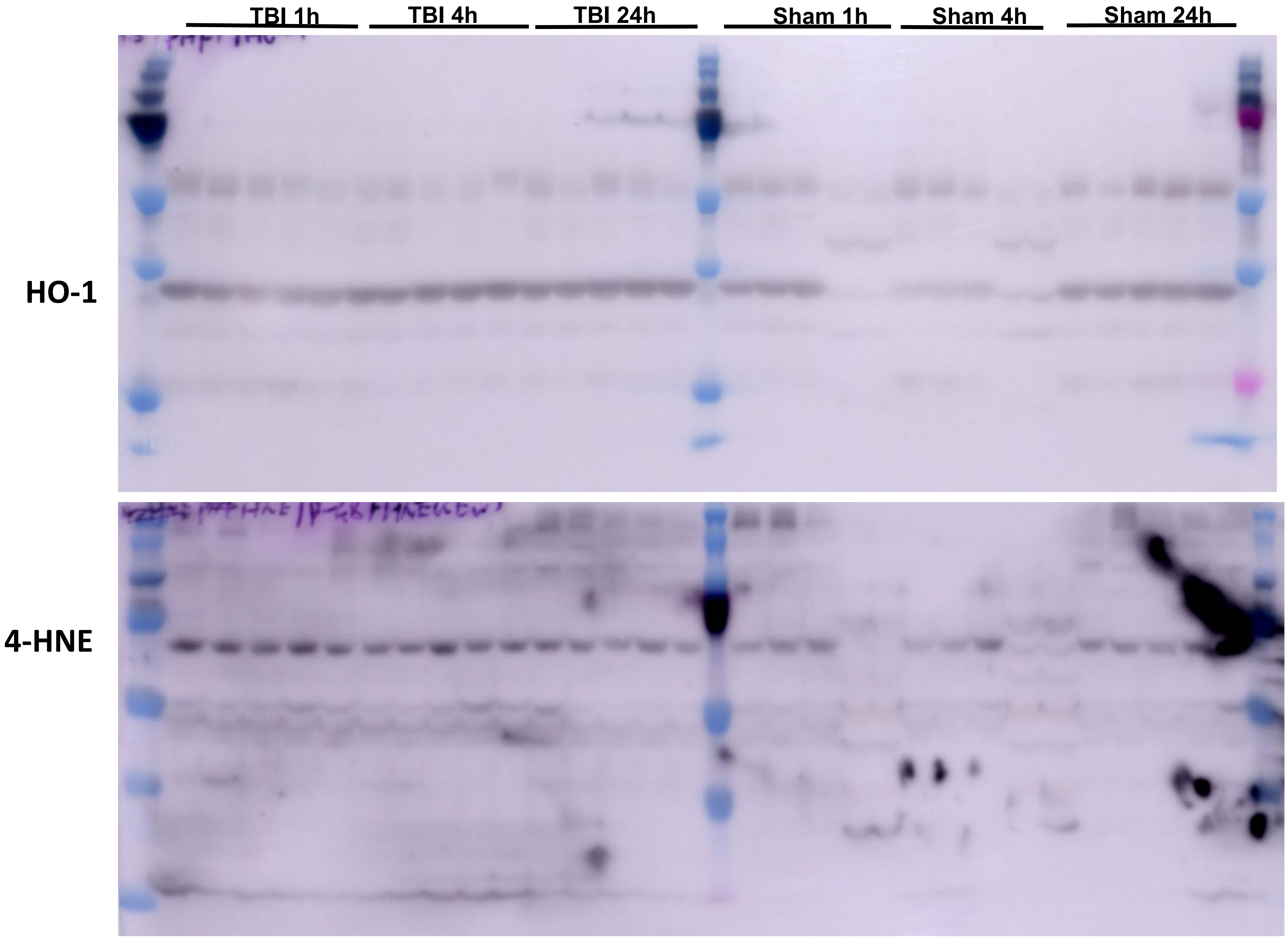
TBI 1h
TBI 4h
TBI 24h
Sham 1h
Sham 4h
Sham 24h
HO-1
4-HNE

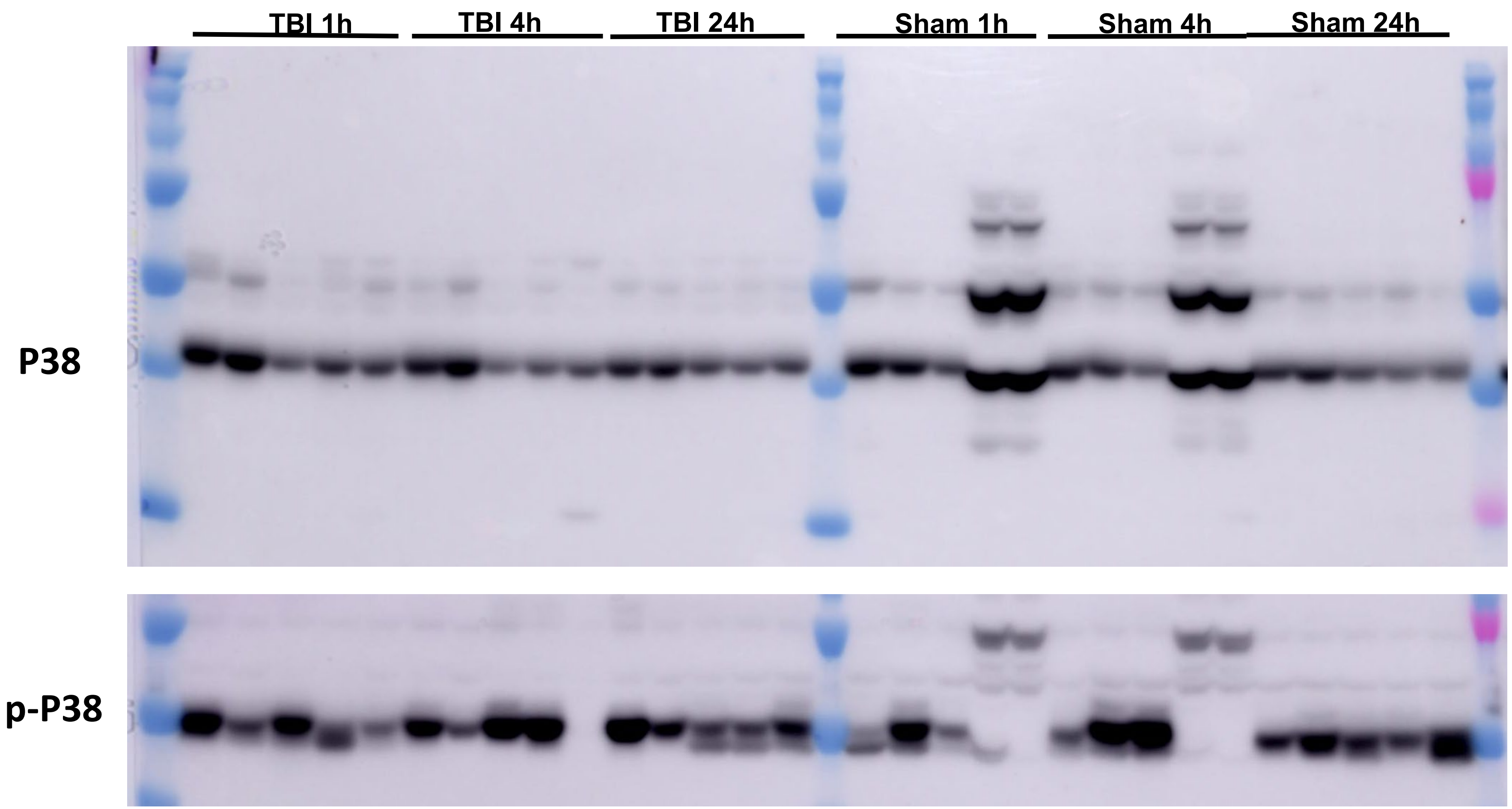
TBI 1h
TBI 4h
TBI 24h
Sham 1h
Sham 4h
Sham 24h
P38
p-P38

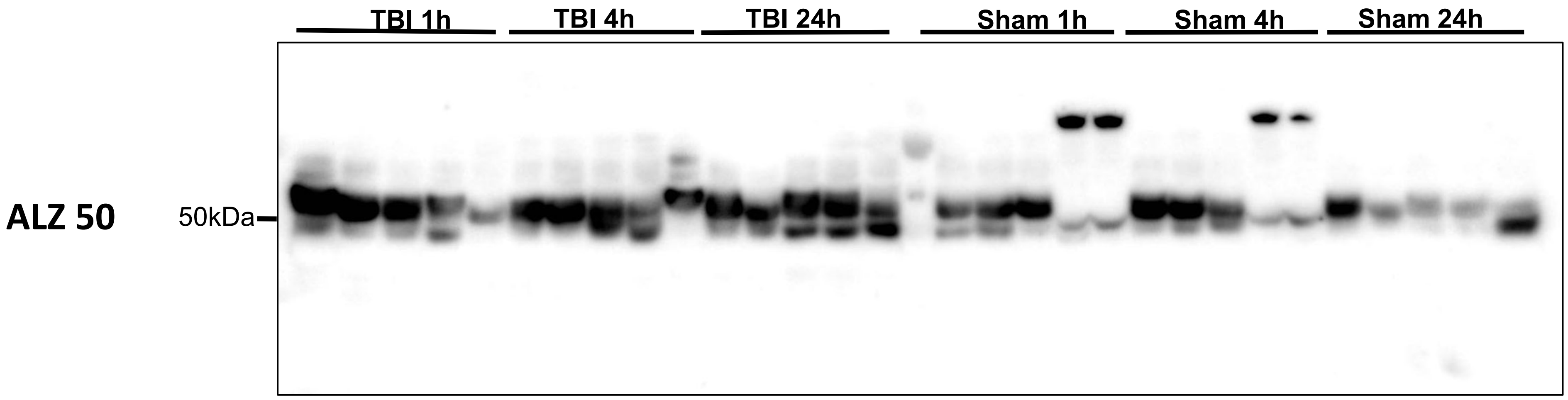

TBI 1h
TBI 4h
TBI 24h
Sham 1h
Sham 4h
Sham 24h
ALZ 50
50kDa
